# Digitally Guided Advanced Prosthodontic Rehabilitation for Post-COVID-19 Mucormycosis Using Patient-Specific Implants: A Case Report

**DOI:** 10.7759/cureus.64729

**Published:** 2024-07-17

**Authors:** Mayuri Surana, Shivsagar Tewary, Nilesh Mishra, Vidit Jain, Pronob Sanyal

**Affiliations:** 1 Department of Prosthodontics, School of Dental Sciences, Krishna Vishwa Vidyapeeth, Malkapur, IND; 2 Department of Oral and Maxillofacial Surgery, School of Dental Sciences, Krishna Vishwa Vidyapeeth, Malkapur, IND

**Keywords:** post-covid-19 mucormycosis, patient-specific implant (psi), bar-retained overdenture, titanium hader bar, maxillary resection

## Abstract

This case report describes the prosthodontic rehabilitation of a middle-aged male patient who underwent bilateral low-level maxillectomy due to post-COVID-19 mucormycosis. The oronasal communication was closed using an anterior base tongue flap. Two patient-specific subperiosteal implants were placed to rehabilitate the patient's bony defect. Using a postsurgical CT scan, a virtually customized subperiosteal titanium framework was created from grade IV titanium alloy. The fabricated framework was implanted over the patient's zygomatic bone bilaterally. Six months later, the right-sided patient-specific implant was infected and had to be surgically removed. After satisfactory healing, an open-tray impression was taken to create a computer-aided design/computer-aided manufacturing titanium Hader bar. An acrylic resin overdenture was then fabricated over this bar. A clasp assembly was fabricated by direct metal laser sintering of cobalt-chromium alloy for additional retention. The metal substructures were incorporated into the overdenture prosthesis to enhance the stability and retention..

This case report unveils an innovative approach to rehabilitating severely compromised maxillary bony defects and impaired oral functioning, offering a viable alternative when traditional reconstruction methods are inadequate. Prosthodontic treatment greatly affects the aesthetics, phonetics, and mastication of the patient, improving the overall quality of life of the patient.

## Introduction

The COVID-19 pandemic has presented numerous challenges to the medical and dental fields, including the rise of secondary infections such as mucormycosis. This opportunistic fungal infection predominantly affects immunocompromised individuals, often leading to severe complications. The maxilla's involvement necessitates aggressive surgical interventions, including resection and debridement, resulting in extensive maxillary defects [1,2].

Such defects present significant rehabilitative challenges, as they require the restoration of not only the missing teeth but also the associated soft tissues and bone, including the hard palate and alveolar ridges. These defects, in turn, affect aesthetics, phonetics, mastication, and deglutition [3]. Traditional reconstruction methods often fail to address these multifaceted issues, particularly in maintaining functionality and aesthetics.

This article explores an innovative approach to the surgical and prosthodontic rehabilitation of a post-COVID-19 mucormycosis patient using advanced computer-aided design (CAD)/computer-aided manufacturing (CAM) technology. Employing patient-specific implants and a titanium bar-retained overdenture offers a promising alternative to conventional techniques.

The present case report highlights the use of cutting-edge digital technology to fabricate precise, customized implants, the titanium bar substructure, and the clasp assembly, which ensures optimal fit and stability of the overdenture prosthesis, ultimately enhancing both the patient's function and aesthetics.

## Case presentation

A 44-year-old male patient reported to the Department of Prosthodontics, School of Dental Sciences, Krishna Vishwa Vidyapeeth, Malkapur, for prosthetic rehabilitation of an acquired maxillary defect secondary to mucormycotic necrosis.

**Figure 1:**
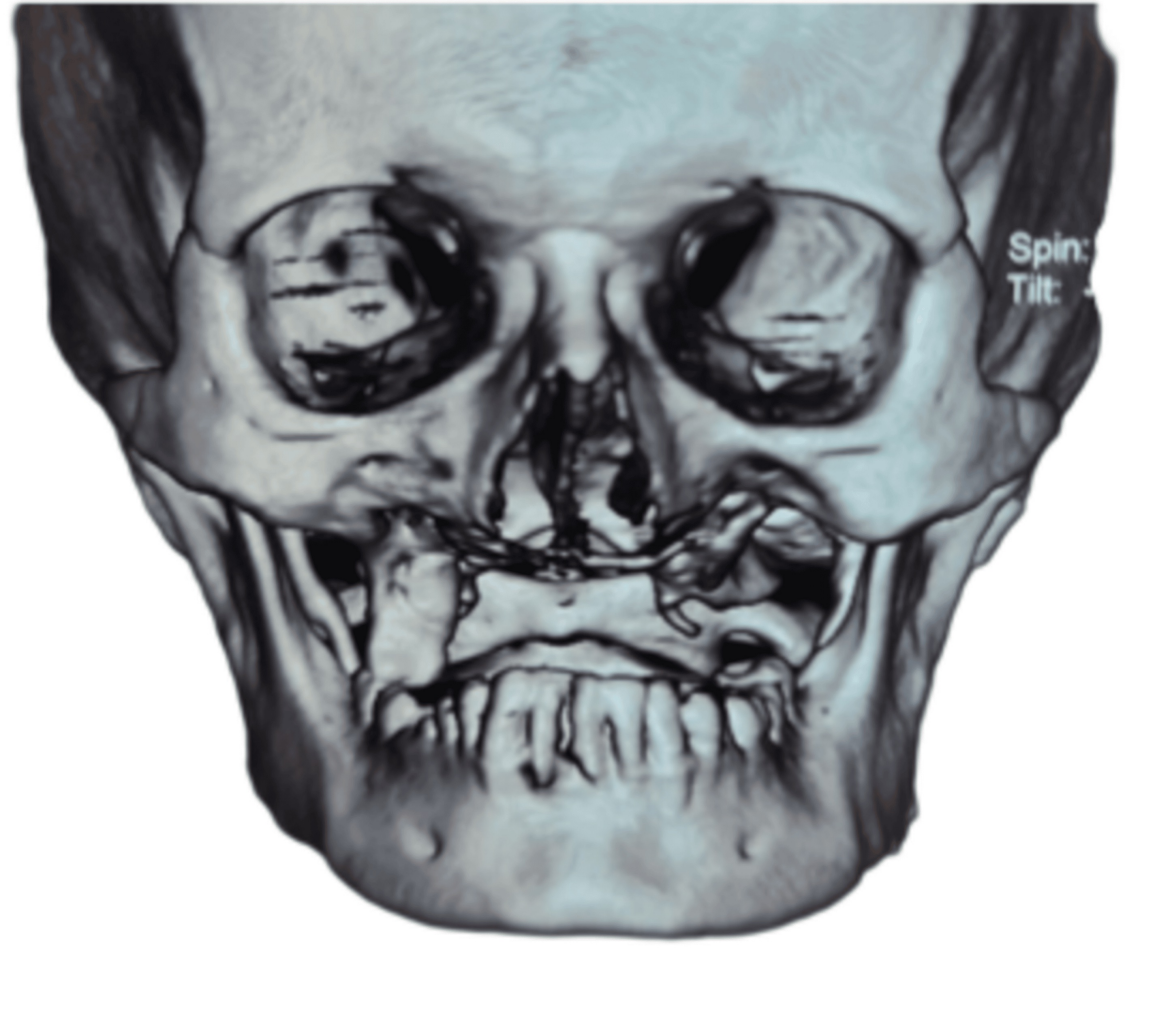
3D CT face for designing patient-specific implants

The patient had undergone various surgical procedures under general anesthesia, such as bilateral subtotal maxillectomy with bilateral maxillary sinus curettage. Secondary closure of the oronasal fistula was done using an anterior base tongue flap detached after 21 days. After six months of the surgery, the patient had aesthetic as well as functional concerns regarding phonetics and mastication. Placing patient-specific implants in the bilateral zygoma was planned to rehabilitate the same. After obtaining the CT scan file (Figure 1), it was imported to form a virtual model, and the designing of the patient-specific implant was done.

**Figure 2:**
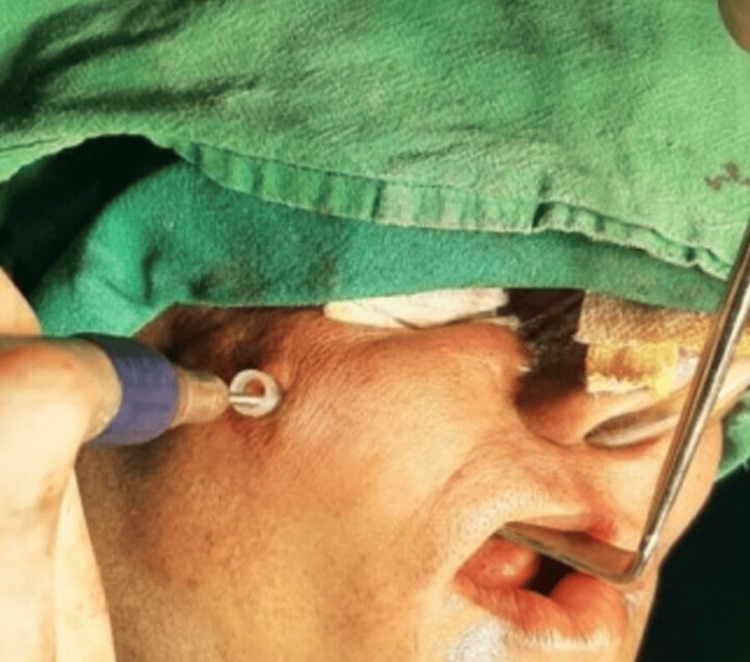
Transbuccal approach for PSI placement PSI: patient-specific implant

These implants were placed over the zygomatic bone through a transbuccal approach on both sides (Figure 2). The patient was kept on follow-up for the next six months, during which the patient showed signs of recurrent infection, such as tenderness and extraoral pus drainage from the right-sided implant region. Antibiotic therapy was prescribed, but the infection still persisted. This led to the decision to remove the right-sided patient-specific implant.

**Figure 3:**
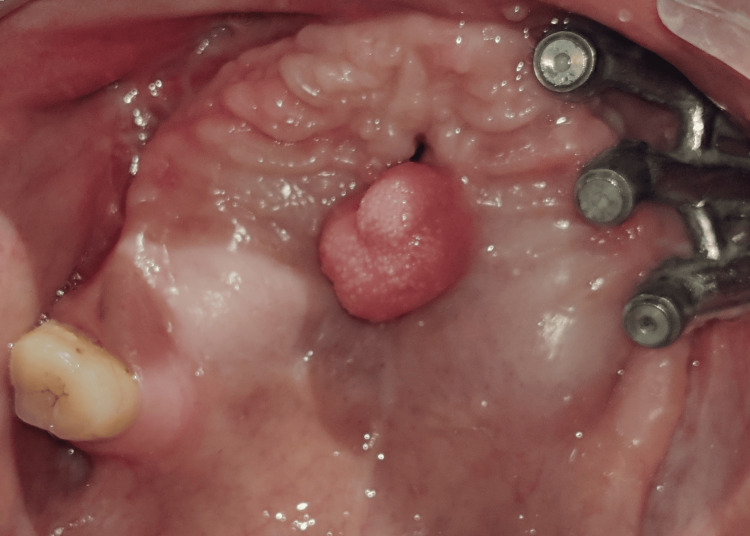
Maxillary occlusal view before treatment

The surgical site was allowed to heal for the next three months before starting the prosthetic treatment. The patient reported to the Department of Prosthodontics with the unilateral patient-specific implant present on the left side, an oronasal fistula, a tongue tissue remnant in the mid-palatal region, and a single natural tooth (right maxillary third molar) on the right side (Figure 3).

The prosthodontic evaluation was done, and the treatment plan was designed according to the clinical presentation of the case. A virtually designed and milled titanium Hader bar was to be fabricated to act as the prosthesis's metal substructure. A bar-retained maxillary overdenture was planned, along with additional retention to be obtained from a clasp assembly fabricated on the remaining natural tooth. Primary impressions were made using irreversible hydrocolloid, poured using dental stone, and the primary casts were obtained. Two-layered modeling wax was adapted on the primary cast to block the undercuts and provide space to accommodate the putty impression material. A self-cure acrylic resin custom tray was prepared with three access holes to expose the open-tray impression copings while recording the implant impression.

**Figure 4:**
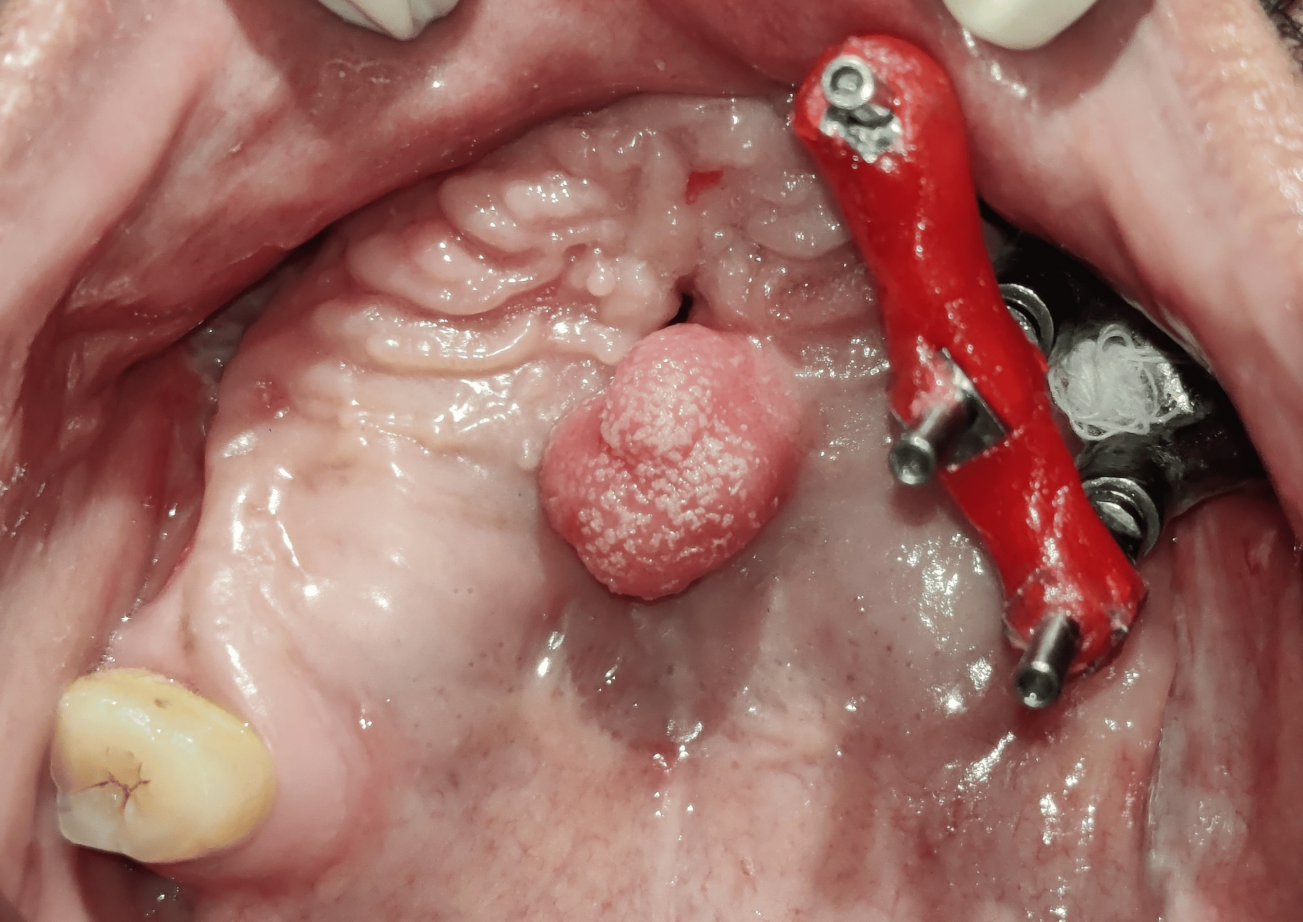
Open-tray impression copings are placed and splinted using pattern resin

**Figure 5:**
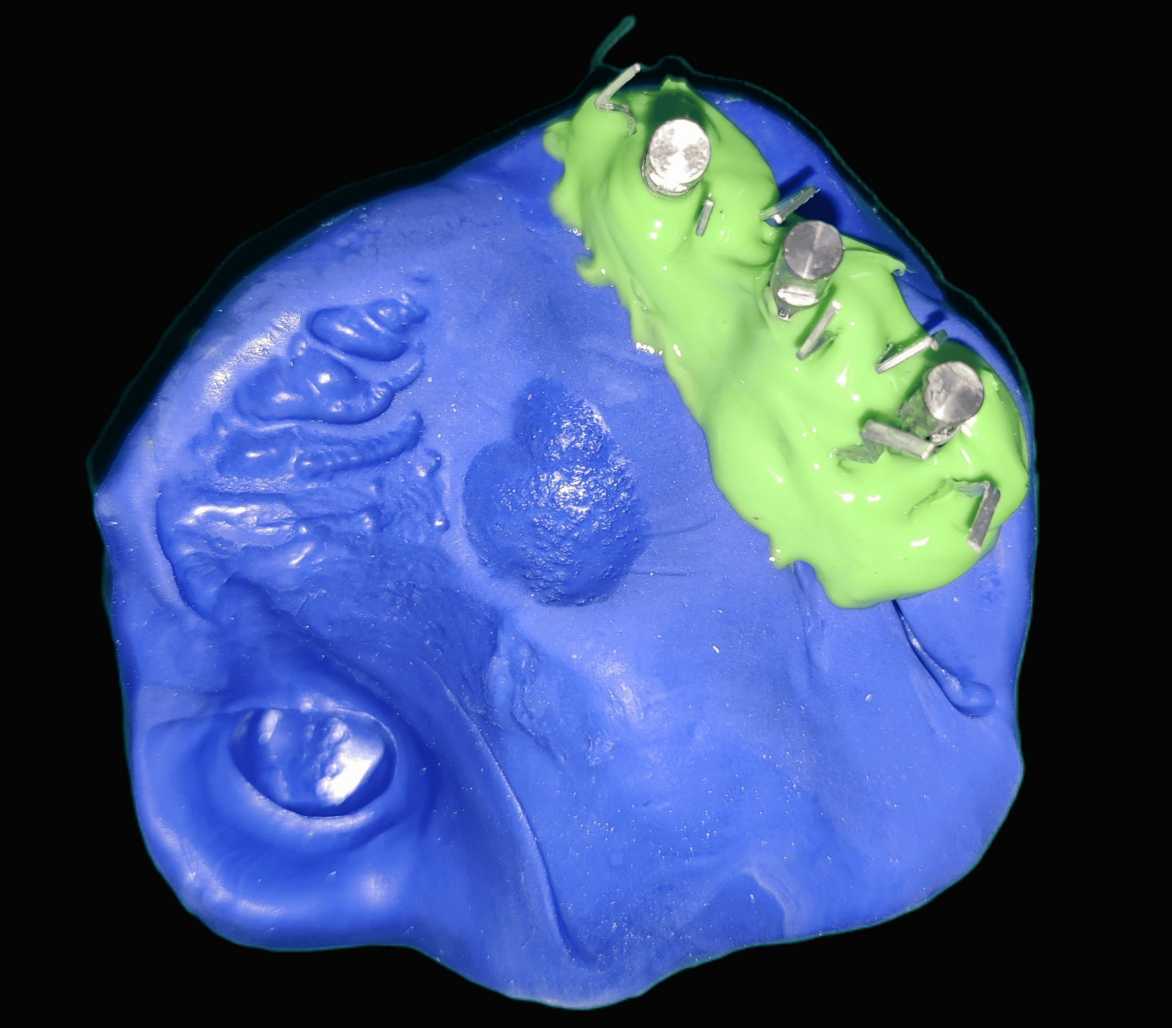
Implant-level putty impression with implant analogs and gingival masking

The open-tray impression copings were splinted using dental floss and stabilized using pattern resin to form a rigid framework for accurate impression-making (Figure 4). Addition-silicone putty was mixed, loaded on the custom tray, placed intraorally, and allowed to set. Once set, the open-tray impression copings were unscrewed, and the impression was retrieved (Figure 5).

**Figure 6:**
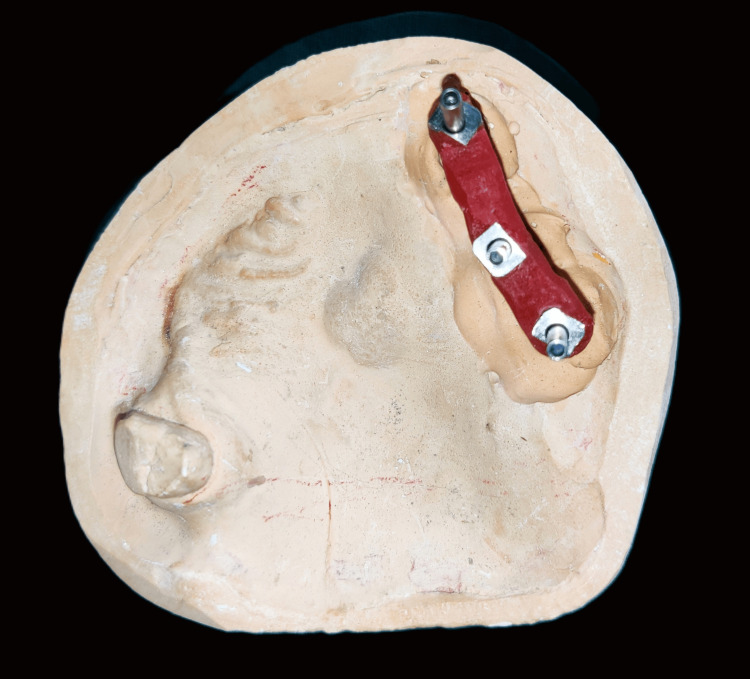
Verified master cast

**Figure 7:**
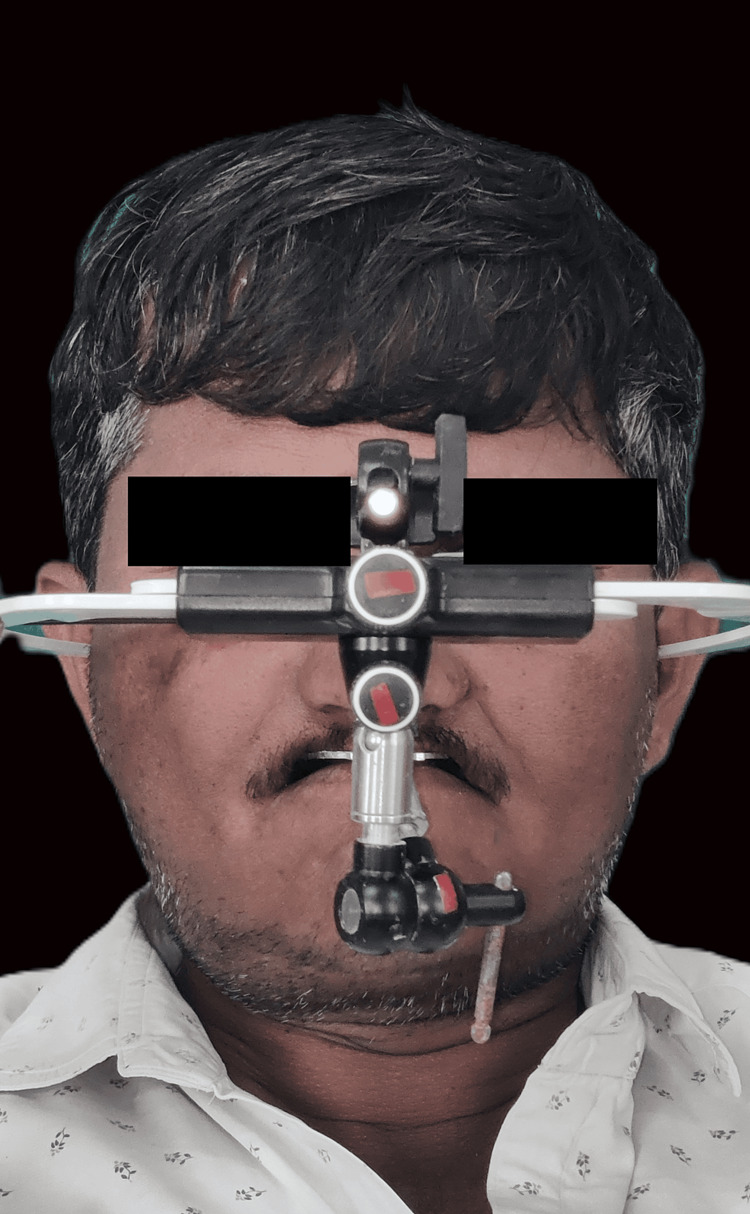
Facebow transfer

Implant analogs were attached to the impression copings, and the die stone master cast was poured. To verify the implant position and orientation, a new pattern resin jig was fabricated on the master cast, and its fit and passivity were checked and verified intraorally. This verified the master cast onto which the prosthesis was going to be fabricated (Figure 6). Maxillomandibular jaw relations were recorded using facebow transfer and mounted on the semiadjustable articulator (Figure 7).

**Figure 8:**
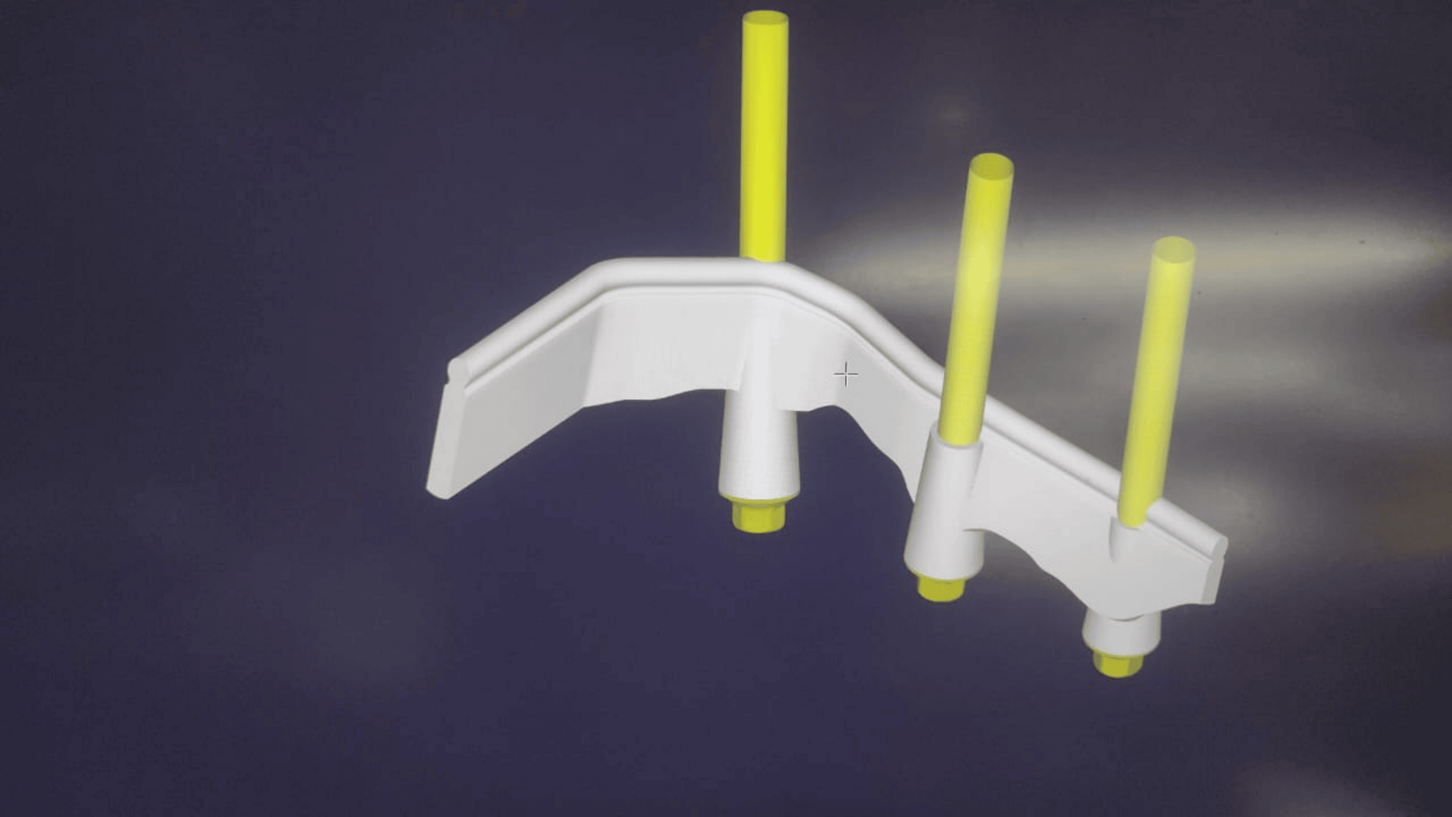
Virtual bar design using the DentalCAD software (exocad GmbH, Darmstadt, Germany)

**Figure 9:**
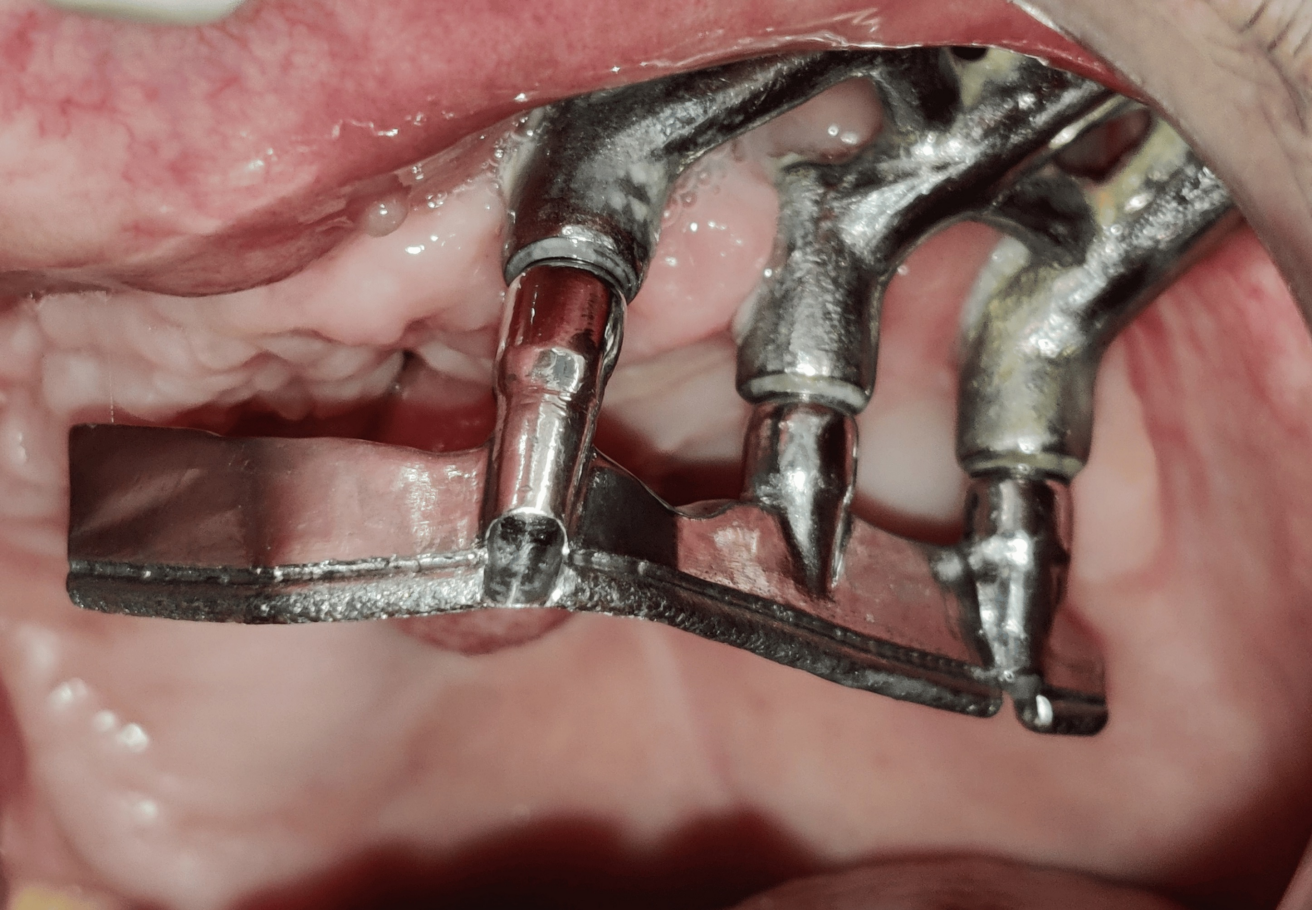
Milled titanium Hader bar

The master cast was scanned using a lab scanner, and a virtual model was created (Figure 8). The titanium Hader bar was designed to cross the midline to provide cross-arch stability, although the length of the cantilevered arm was kept limited to prevent mechanical failures. This design was then finalized, and the titanium bar was milled from the titanium disc. This bar was placed intraorally, and the passivity of fit was confirmed using the Sheffield test (Figure 9).

**Figure 10:**
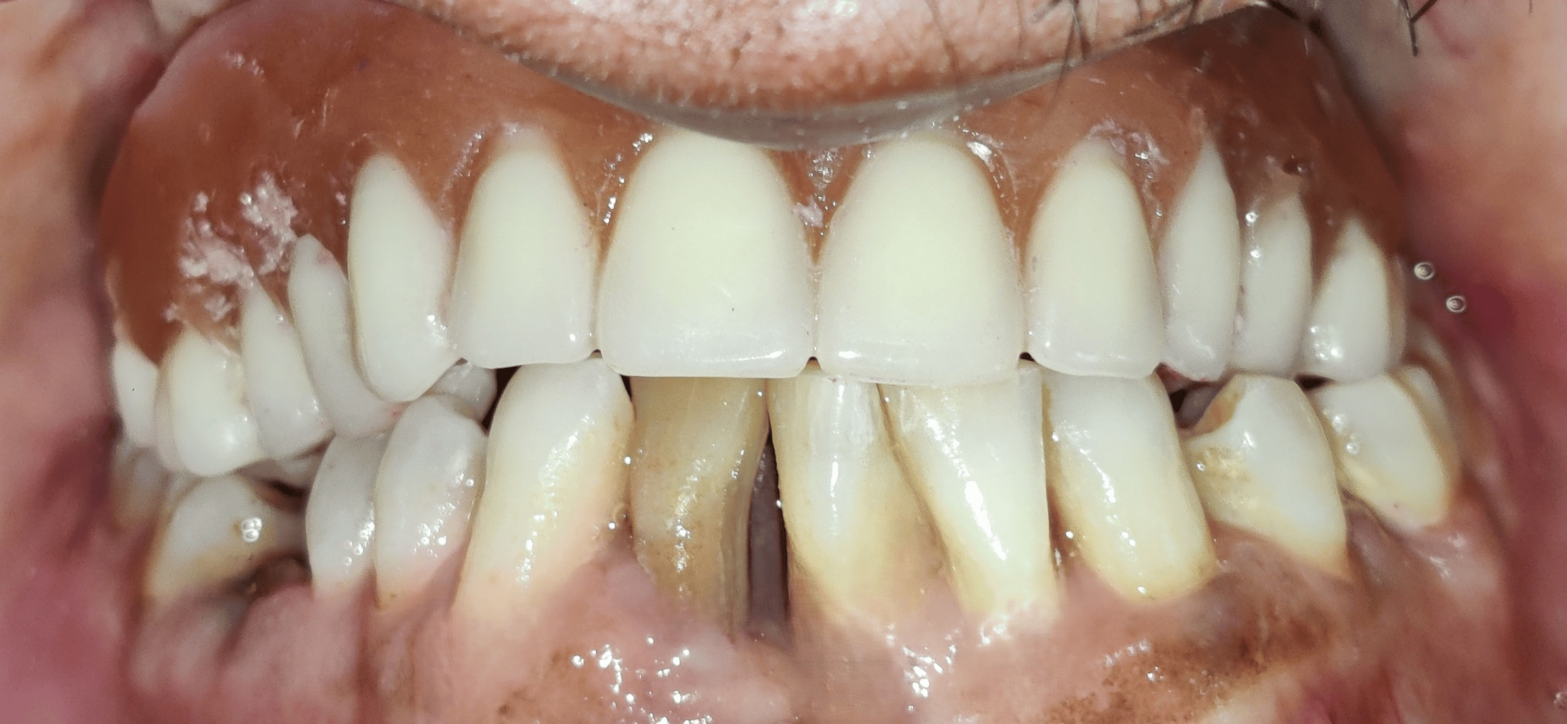
Trial prosthesis

**Figure 11:**
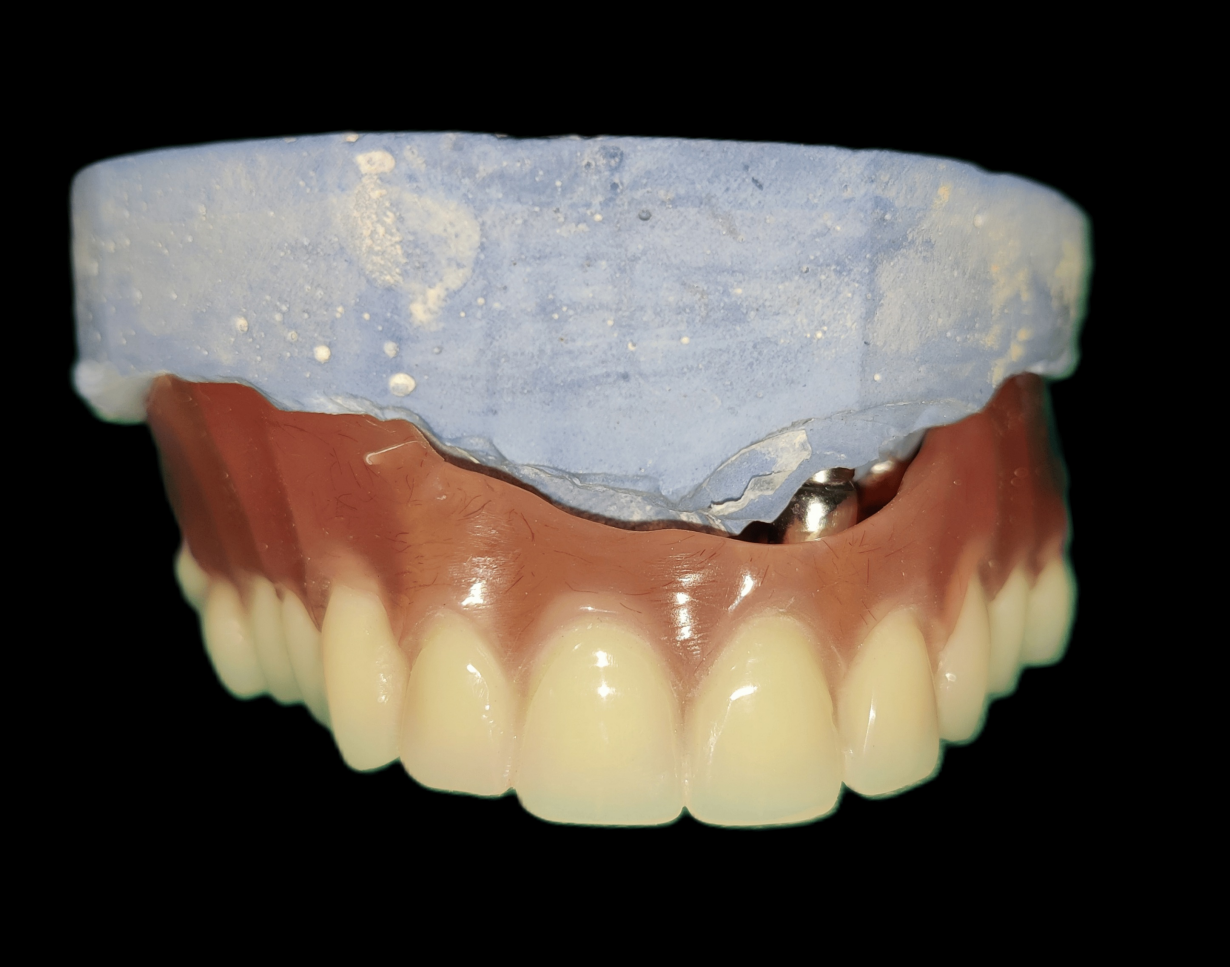
Heat cure acrylic overdenture

**Figure 12:**
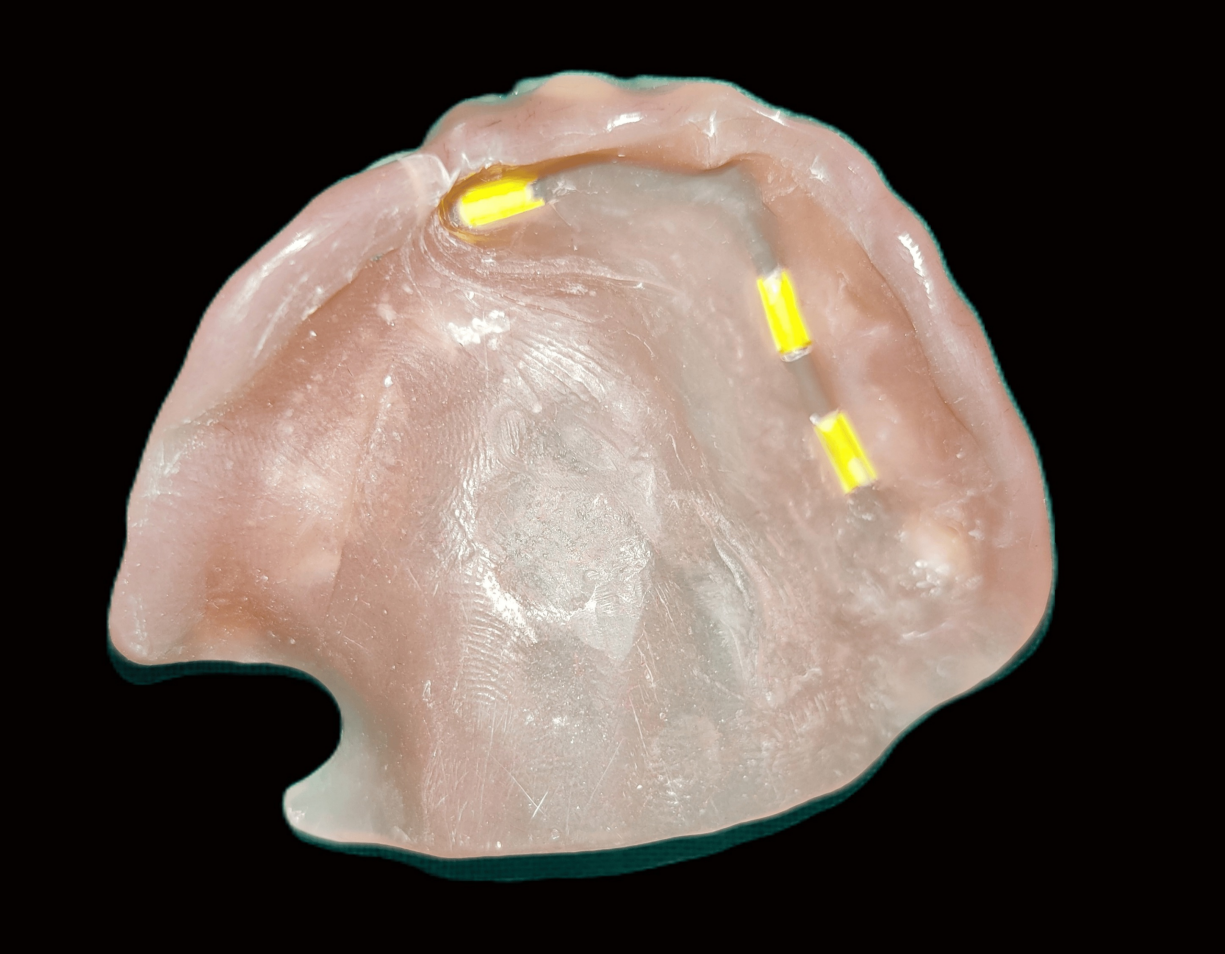
Retentive clips on intaglio surface after lab pickup

The next step was the fabrication of the bar-retained overdenture. A record base was fabricated over the bar placed on the cast, and then teeth were arranged over it. The trial prosthesis was checked for proper esthetics and phonetics, and bilateral occlusion was also verified in the final try-in appointment (Figure 10). After the try-in appointment, acrylization of the trial prosthesis was performed, and the heat cure acrylic resin prosthesis was fabricated (Figure 11). It was finished and polished, and the retentive clips' lab pick-up was performed by blocking the undercuts on the cast (Figure 12).

**Figure 13:**
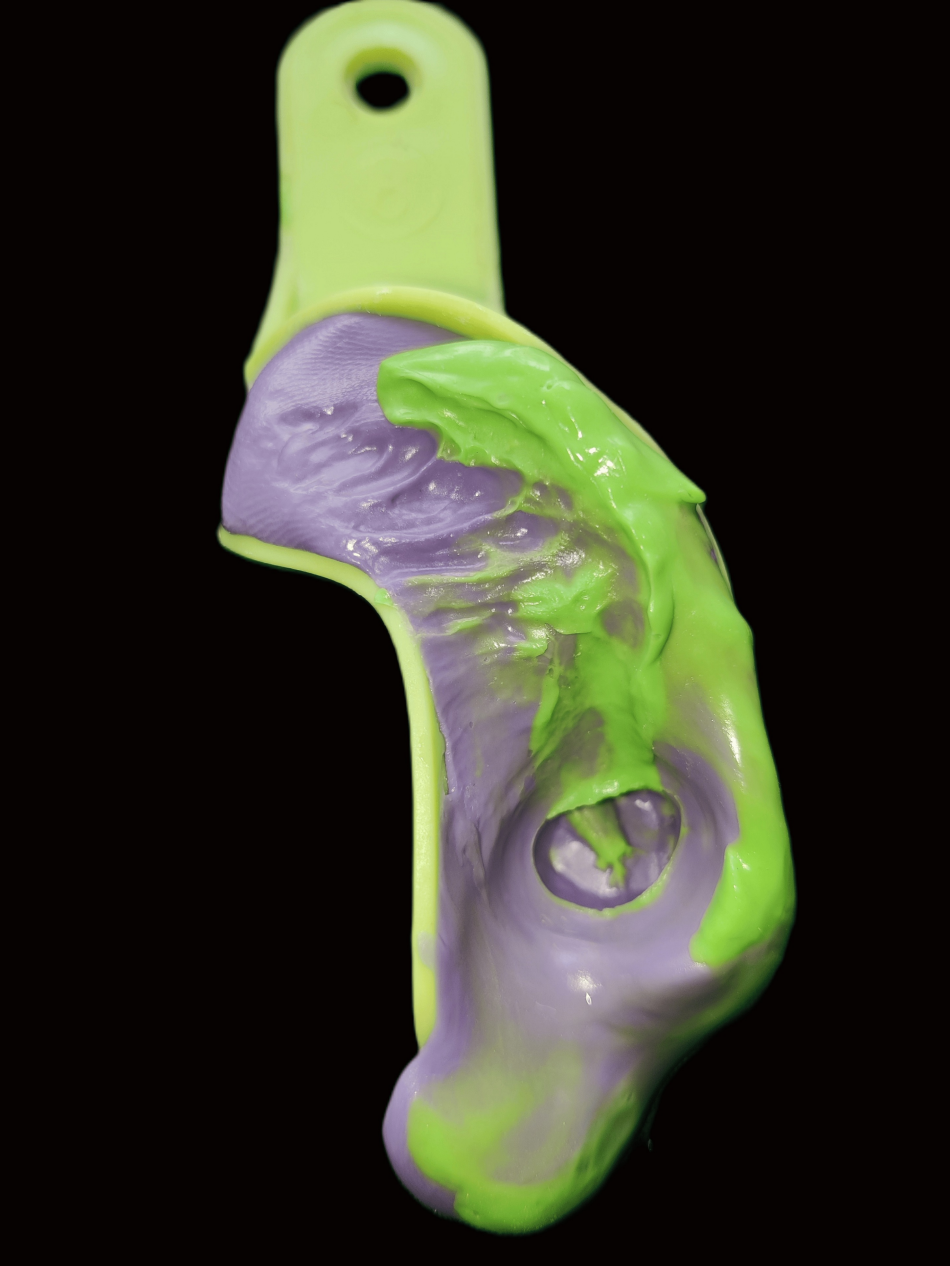
Putty impression of prepared rest seat

**Figure 14:**
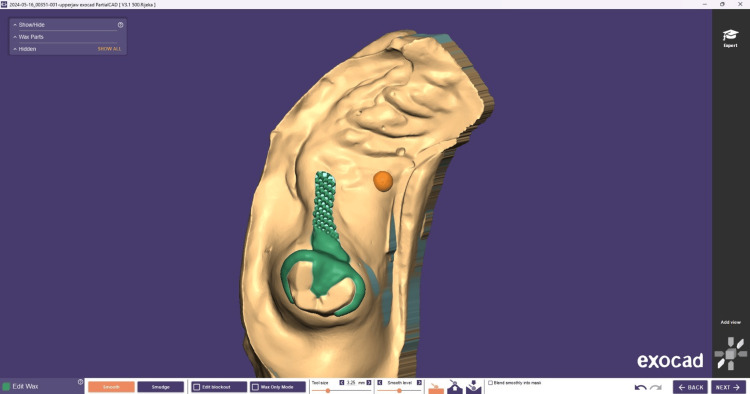
Virtual design of clasp assembly using the DentalCAD software (exocad GmbH, Darmstadt, Germany)

**Figure 15:**
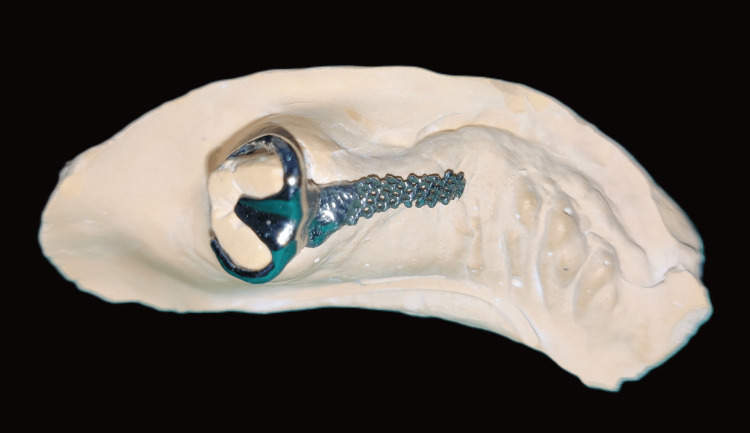
DMLS cobalt-chromium clasp assembly DMLS: direct metal laser sintering

The last retentive feature to be added to the prosthesis was the natural tooth-supported clasp assembly. For its fabrication, a rest seat was prepared on the third molar, having a buccolingual width of one-third of that of the tooth and about half that of the tooth mesiolingually. A putty impression was made of the prepared tooth, and a die stone cast was poured (Figure 13). This cast was scanned using a lab scanner to make the virtual model over which the clasp assembly was digitally designed (Figure 14). This design was then milled using direct metal laser sintering of the cobalt-chromium alloy to fabricate the clasp assembly. This clasp assembly comprised a retentive arm adapted on the buccal surface, a reciprocal arm on the palatal surface, a rest on the occlusal surface, and an extension of the metal mesh framework to attach to the overdenture (Figure 15).

**Figure 16:**
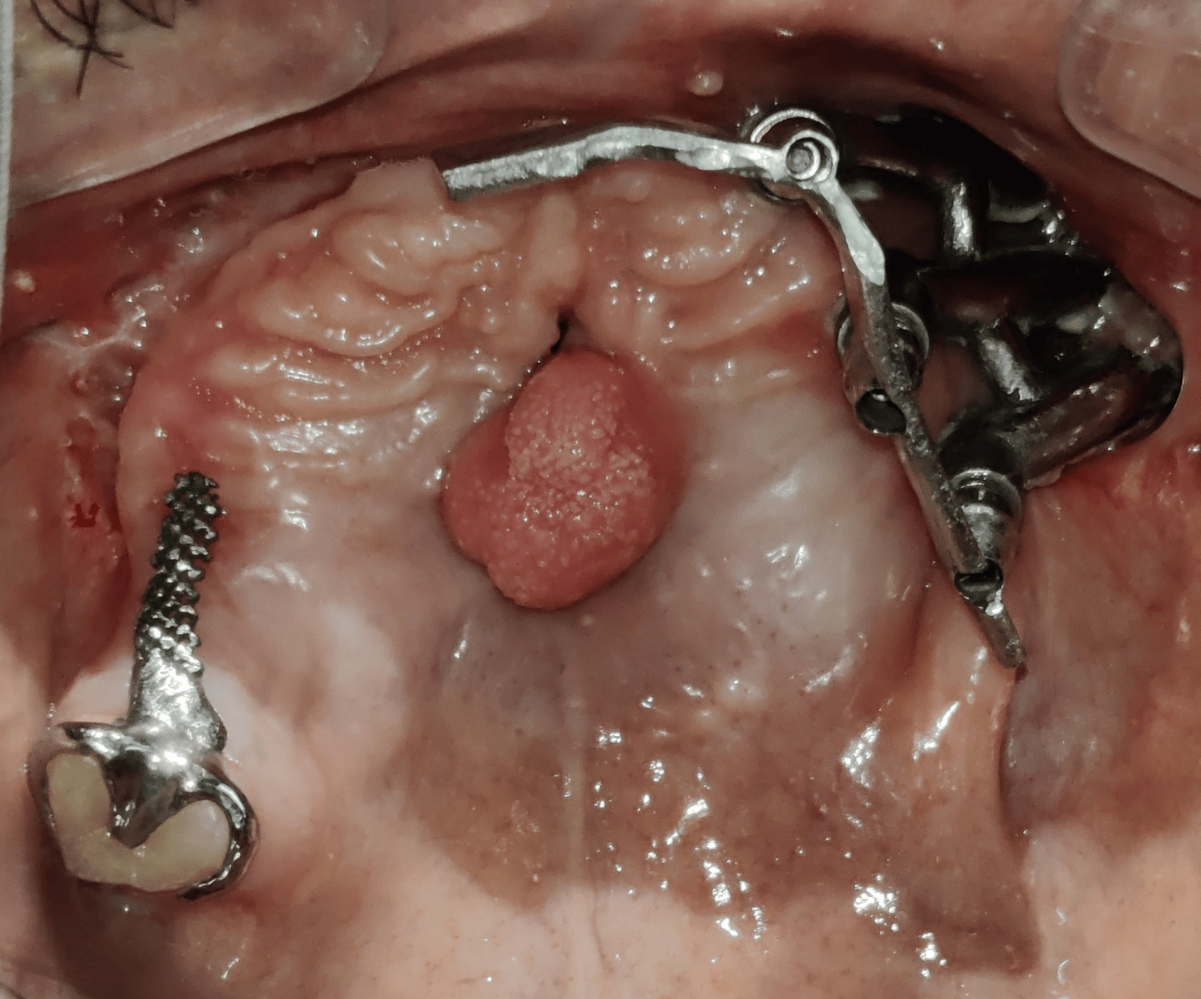
Metal substructure placed for clinical pickup

**Figure 17:**
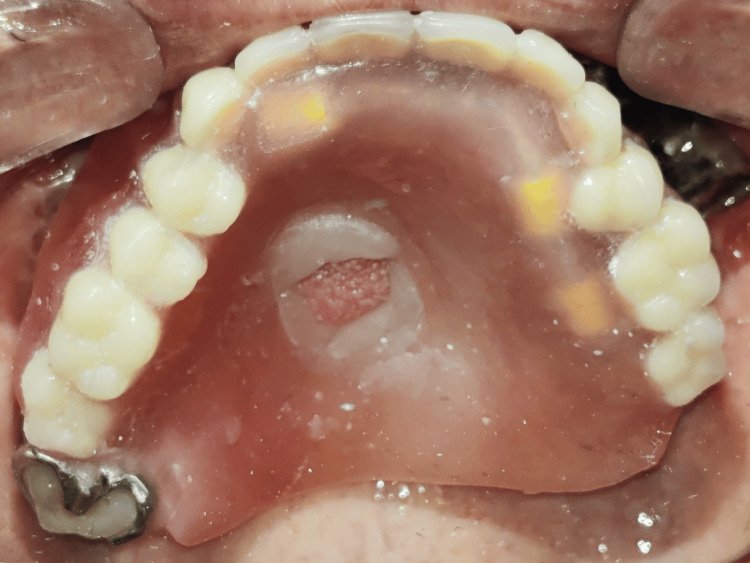
Overdenture with a soft liner to relieve tongue tissue remnant

**Figure 18:**
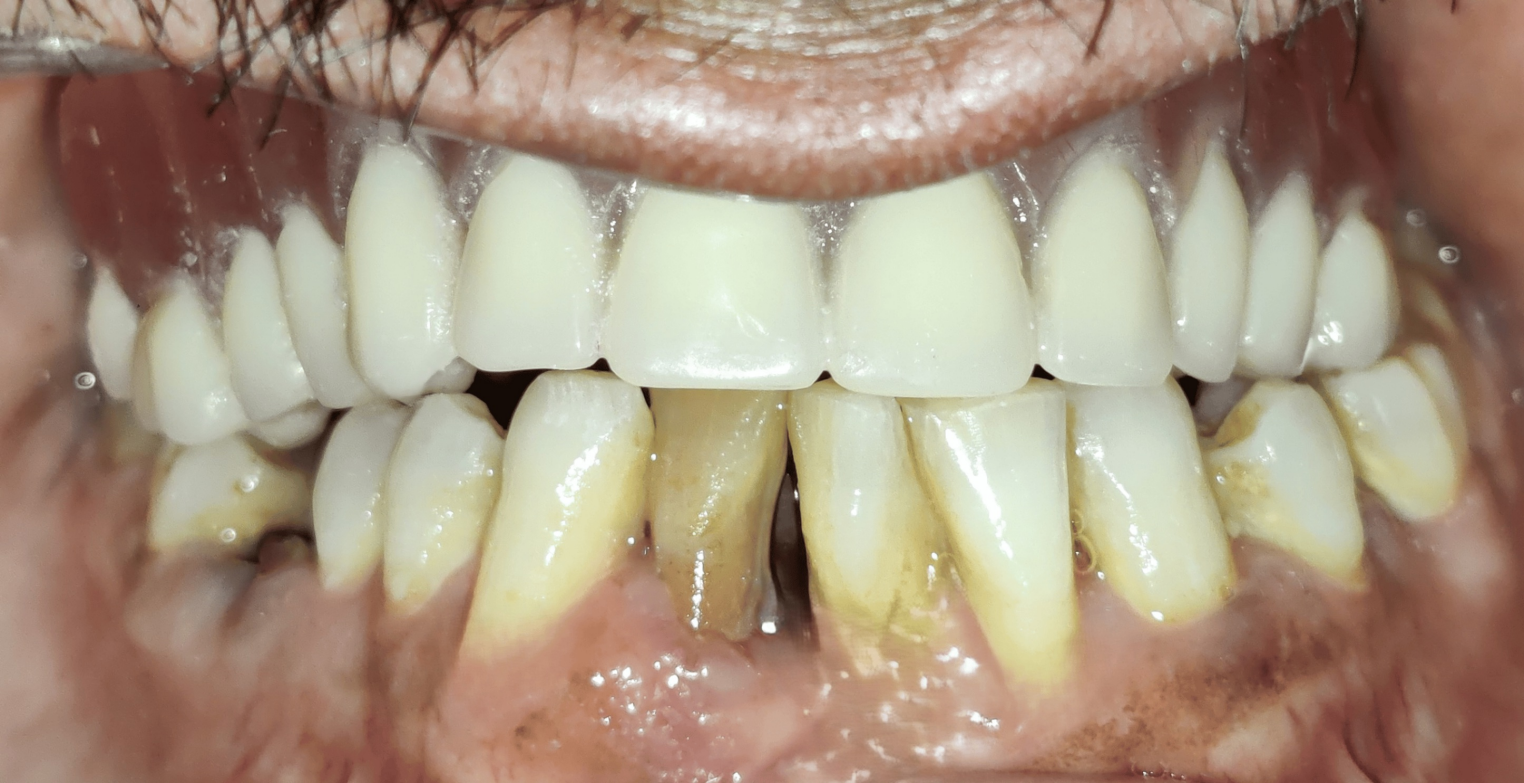
Final prosthesis with verified aesthetics, phonetics, and occlusion

The clasp assembly was placed intraorally, the adjacent tissues were isolated, self-cure acrylic was loaded into the overdenture, and the clinical pickup of the clasp assembly was done (Figure 16). The area was examined, finished, and polished to ensure no sharp projections or irregularities were present on the intaglio and the cameo surface of the denture. The palatal plate of the denture was cut out because of the hindrance of tongue tissue remnant in the mid-palatal region, which prevented tissue contact with the rest of the prosthesis. A soft liner was used to line the cutout area to prevent damage to the tissue during insertion and removal and to prevent the leakage of fluid and food regurgitation through the oronasal fistula (Figure 17). The final prosthesis was inserted into the patient’s mouth, and the esthetics, phonetics, and bilateral occlusion were verified (Figure 18).

**Figure 19:**
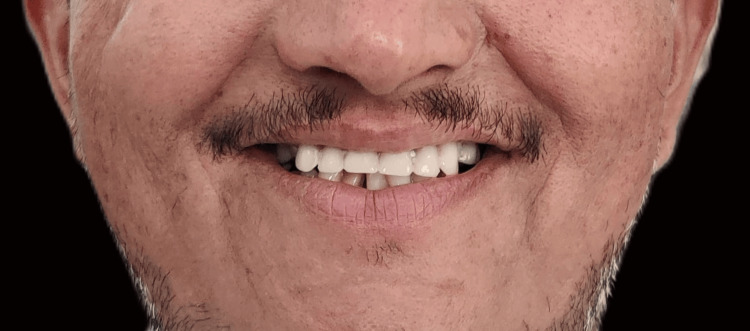
Posttreatment front-smiling photograph

The patient was given hygiene and maintenance instructions and trained to insert and remove the prosthesis. The confident smile on the patient’s face determined the clinical success of the prosthetic rehabilitation (Figure 19).

The follow-up visits were after 15, 30, 45, 60, and 90 days. During these visits, the occlusal refinements, speech quality, and peripheral seal were evaluated. After the last follow-up period, the patient was satisfied with the prosthesis's routine usage.

## Discussion

When a patient undergoes extensive surgery such as maxillectomy, there are various challenges that the patient faces, such as oronasal fistula, which leads to fluid and food regurgitation and alters the phonetics of the patient; loss of support for cheek and lips due to midfacial defects of the bone and soft tissues thus compromising the esthetics of the patient; functional impairment of speech due to loss of alveolus and loss of teeth; and masticatory difficulties due to loss of teeth and presence of oronasal fistula [4].

The surgeon or the operator also faces various challenges when reconstructing extensive defects like these. This may include the presence of oronasal communication, which must be separated. There may also be a lack of available tissue to obtain support for rehabilitation. Another challenge is the need to minimize the donor site morbidity. In addition, the local vascular bed may be compromised due to bone necrosis caused by the infection. Also, the systemic physical condition of the patient may be compromised due to previous surgical procedures and medications administered [3].

Reconstruction goals include adequate oronasal separation, restoration of the alveolar anatomy, preservation of speech and mastication, and provision of facial height and midfacial projection [5]. Prosthetic rehabilitation in such cases may include various treatment options, such as a fixed implant-supported porcelain fused to metal (PFM) prosthesis, a bar-retained fixed hybrid prosthesis, a titanium Hader bar-retained overdenture, or a magnetic attachment-retained overdenture [6]. The milled titanium Hader bar provided us with the benefits of precision, customization, strength, durability, lightweight, and compatibility with modern dental technology [7].

The benefits of the bar-supported overdenture include improved stability and retention, better force distribution, enhanced aesthetics, ease of repair or replacement, lightweight, and better comfort [8]. In the present case, a titanium Hader bar-retained overdenture was opted as the best suited treatment modality because of various factors, such as the presence of a unilateral patient-specific implant where full arch fixed prosthesis, either PFM or hybrid type, was contraindicated due to the need for the cantilever of the bar, which may lead to mechanical complications [9].

The presence of an oronasal fistula and the need for its closure inclined toward the use of an overdenture prosthesis, which would both provide bilateral occlusion as well as be useful in blocking the oronasal fistula [10]. The clasp assembly enhanced retention, increased stability, enabled even force distribution, and provided snug and secure fit and durability to the prosthesis [11]. The drawbacks of the prosthesis include the attrition of the acrylic teeth, the wear of the acrylic, and the fracture of the components [12].

## Conclusions

Mucormycosis causing orofacial defects greatly affects the quality of life of the patient. The advanced prosthodontic rehabilitation using advanced radiography, digital technology, patient-specific implants, and a titanium bar-retained overdenture demonstrated remarkable success. Titanium-based 3D patient-specific implants provide an innovative solution to rehabilitate such extensive defects. The progressive approaches employed, such as innovative prosthesis design, CAD/CAM technology, additional retention strategy utilizing titanium Hader bar, and cobalt-chromium clasp assembly, along with the use of removable prosthesis, were pivotal in overcoming the obstacles encountered during prosthetic rehabilitation. The patient reported high satisfaction with the treatment, noting significant improvements in comfort, function, and overall quality of life. This novel method underscores the potential of digital prosthodontics and custom retentive solutions in addressing complex rehabilitation cases, paving the way for future advancements in the field.
